# Dinoflagellate mRNA is pervasively modified with m^1^A

**DOI:** 10.1038/s44319-024-00263-x

**Published:** 2024-09-20

**Authors:** Jianheng Fox Liu, Samie R Jaffrey

**Affiliations:** Department of Pharmacology, Weill Cornell Medicine, Cornell University, New York, NY 10065 USA

**Keywords:** Chromatin, Transcription & Genomics, RNA Biology

## Abstract

Unlike the scarce presence in typical eukaryotes, m^1^A is prevalent in dinoflagellate mRNA, m^1^A levels correlate with the expression of metabolism-related genes and respond to nitrogen starvation.

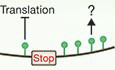

## Teaser

*N*^1^-methyladenosine (m^1^A) is a very rare RNA modification in mammalian mRNA, which occurs in sequences resembling the T-loop of tRNAs and may not have significant effects on gene expression. A new study in this issue now reports that m^1^A is abundant and dynamic in the 3′UTR of mRNAs of dinoflagellates, and could have an important role in post-transcriptional gene expression regulation in these organisms.

Nucleotide modification is an important mechanism of post-transcriptional gene regulation. To date, over 170 types of nucleotide modifications have been found in cellular RNA (Wiener and Schwartz, 2021). Most of the RNA modifications occur exclusively in rRNA and tRNA, while only a few nucleotide modifications are observed in mRNA, such as *N*^6^-methyladenosine (m^6^A), pseudouridine (Ψ), and 5-methylcytidine (m^5^C). *N*^1^-methyladenosine (m^1^A) was also reported in mammalian mRNA, with original reports describing several thousand m^1^A-containing transcripts (Dominissini et al, 2016; Li et al, 2016). However, subsequent studies showed that the m^1^A-binding antibody used to isolate m^1^A-containing mRNA fragments was not specific (Grozhik et al, 2019; Safra et al, 2017). In part, this was evident by reverse transcribing the mRNA fragments bound by the m^1^A antibody. The resulting cDNA failed to show misincorporations that are normally induced by m^1^A during reverse transcription (Safra et al, 2017). As a result of these subsequent studies, m^1^A was found to be a rare modification in mammalian mRNA. However, recent evidence from Li et al (2024) demonstrates that m^1^A is abundant and might be involved in post-transcriptional gene expression in dinoflagellates, a group of unicellular eukaryotes commonly found in aquatic ecosystems.

Li et al (2024) first quantified m^1^A stoichiometry in the dinoflagellate species *Amphidinium carterae* (*A. carterae*) by mass spectrometry. The authors found that m^1^A is surprisingly abundant in *A. carterae* mRNA, with up to 3.05% adenosines being modified. This is remarkably high for an mRNA modification. In comparison, m^6^A, the most prevalent mRNA modification in many species, normally accounts for ~0.2–0.6% of the adenosines (Sun et al, 2023). This phenomenon was also observed in other dinoflagellate species such as *Crypthecodinium Cohnii* and *Symbiodinium* sp., suggesting that m^1^A is generally abundant in dinoflagellate mRNA.

**Figure 1:**
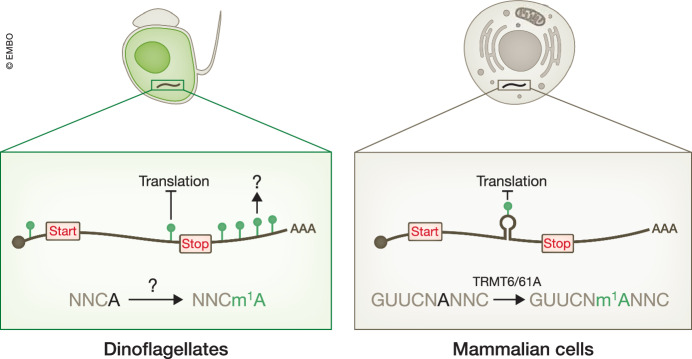
m^1^A distribution is different between dinoflagellates and mammalian cells. In dinoflagellate mRNA (left), m^1^A is abundant and specifically enriched in the 3′UTR. m^1^A is formed by unknown methyltransferase(s) which catalyze m^1^A formation in an NNCA motif. m^1^A in dinoflagellate mRNA, especially in the CDS region, likely represses translation. The function of m^1^A in the 3′UTR is not clear. In contrast, m^1^A is rare in mammalian mRNA (right), and is deposited by the TRMT6/61A complex, which prefers tRNA T-loop-like sequences. m^1^A in the CDS of mammalian mRNA is known to inhibit translation.

Li et al (2024) then explored the distribution of m^1^A across the transcriptome. They used m^1^A MeRIP-seq (m^1^A-specific methylated RNA immunoprecipitation) (Li et al, 2016), which enriches m^1^A-containing transcript fragments and thus reveals m^1^A peaks. They also used m^1^A-seq-TGIRT, which involves performing reverse transcription of cellular mRNA with the TGIRT reverse transcriptase, which is highly efficient at producing misincorporations in cDNA upon encountering m^1^A (Safra et al, 2017). Li et al (2024) established the transcriptomic sites that contained m^1^A peaks and misincorporations to map m^1^A across the *A. carterae* transcriptome. Intriguingly, they found that ~80% of m^1^A sites were in the 3′ untranslated regions (UTRs) (Fig. 1). Because this pattern was reproduced by both m^1^A MeRIP-seq and m^1^A-seq-TGIRT, this distribution of m^1^A is likely correct.

In mammalian cells, the TRMT6/61A complex preferentially methylates adenosines in a GUUCNANNC (A = m^1^A) motif in short stem-loops resembling the tRNA T-loop (Safra et al, 2017). However, the authors found that the m^1^A in *A. carterae* mRNA is associated with an NNCA (A = m^1^A) motif, which is notably different from the tRNA T-loop. Furthermore, the authors found that the dinoflagellate TRMT6/61A complex homolog does not methylate mRNA in vitro. Therefore, mRNA m^1^A in dinoflagellate may be deposited by a different unknown methyltransferase(s).

Dinoflagellates rely heavily on post-transcriptional gene regulation due to their limited ability to alter transcript expression levels in response to environmental changes (Roy and Morse, 2013; Zaheri and Morse, 2022). Li et al (2024) thus hypothesized that m^1^A might play an important role in enhancing the flexibility of gene expression of dinoflagellates.

To understand the function of m^1^A, Li et al (2024) began by analyzing m^1^A in *A. carterae* in normal growth conditions. The authors found that mRNAs containing m^1^A tended to have higher transcript expression levels, shorter poly(A) tails, and more stable 3′UTR secondary structures. Additionally, the authors observed that mRNAs with m^1^A, particularly those with m^1^A in the CDS region, tend to have lower translation efficiency. This observation is consistent with previous findings in mammalian cells, and might be explained by the hypothesis that the N1-methyl of m^1^A disrupts base pairing between mRNA and tRNA (Safra et al, 2017).

Li et al (2024) then asked if levels of m^1^A are dynamic, which can suggest that m^1^A may be regulated to influence gene expression. Because many m^1^A-modified mRNAs encode proteins related to nitrogen metabolism, the authors tested culturing dinoflagellates in media lacking nitrogen sources (“N-depletion”). N-depletion causes global translational repression, but the authors also found that m^1^A levels were generally reduced throughout the transcriptome. The authors found that mRNAs that normally contain m^1^A became de-repressed, potentially due to the loss of m^1^A, allowing these mRNAs to have relatively increased translation. The authors found a small subset of mRNAs to exhibit increased m^1^A after N-depletion. These mRNAs showed enhanced translational suppression, consistent with the idea of combined translation-repressive effects of m^1^A and N-depletion. Since m^1^A-modified mRNAs are associated with nitrogen metabolism, the data suggest that this dynamic control of m^1^A may contribute to adaptive responses to nitrogen depletion.

Taken together, this study by Li et al (2024) provides evidence that m^1^A can be abundant and dynamic in mRNA, and might influence gene expression. Moving forward, it will be important to identify the methyltransferase(s) responsible for m^1^A deposition in dinoflagellates. This will help to identify pathways that control m^1^A levels in mRNAs. It will be very important to understand why m^1^A is specially enriched in the 3′UTR, which may be caused by targeting of the methyltransferase to this transcript region. Alternatively, m^1^A in the coding region may stall ribosomes and lead to mRNA depletion, thus reducing the prevalence of mRNAs with m^1^A in the coding sequence. Although this study suggests that m^1^A inhibits translation, it is worth mentioning that the majority of m^1^A sites are in the 3′UTR. These m^1^A sites should have smaller effects on translation inhibition since m^1^A in the 3′UTR would not encounter the ribosome to trigger translation repression. It will be important to establish the functions of m^1^A in the 3′UTR, which might contribute to RNA stability or may influence the binding of RNA-binding proteins.
